# Trauma‐Informed Clinical Supervision for Nurses and Midwives: An Integrative Review of the Literature

**DOI:** 10.1111/inm.70381

**Published:** 2026-10-22

**Authors:** Debra Klages, Eric Lim, Martina Costello, Debra Carnes, Katrina Angus, Kim Usher

**Affiliations:** ^1^ University of New England Armidale New South Wales Australia; ^2^ University of Newcastle Newcastle New South Wales Australia; ^3^ Murdoch University Perth Western Australia Australia; ^4^ National Coalition of Independent Scholars Glastonbury Connecticut USA; ^5^ Queensland Health Rockhampton Queensland Australia

**Keywords:** clinical supervision, integrative review, midwives, nurses, trauma‐informed care

## Abstract

Nurses and midwives deliver 24/7 care for complex biopsychosocial needs across the lifespan. The global shortage of nurses and midwives is a challenge for healthcare organisations, placing additional stress and symptoms of trauma on those who remain. Trauma‐informed care is an organisational framework that embeds trauma awareness into healthcare policies and practices to prevent re‐traumatisation and promote the well‐being of patients and providers. Grounded in six core principles, it addresses the multifaceted effects of trauma in healthcare. Clinical supervision supports practitioner development and wellbeing and may benefit nurses and midwives who face vicarious trauma. Although trauma‐informed clinical supervision is established in allied health professions, research in nursing and midwifery is limited. As nurses and midwives also encounter traumatic experiences, further research into trauma‐informed clinical supervision is needed. This review investigated trauma‐informed clinical supervision in nursing and midwifery, asking the question: ‘Can the six principles of trauma‐informed care be integrated into clinical supervision for nurses and midwives?’ An integrative review was employed, identifying seven published articles for inclusion which were then analysed using directed qualitative content analysis. Descriptive knowledge and understanding of the six principles of trauma‐informed care (safety, trustworthiness and transparency, peer support, collaboration and mutuality, empowerment, voice and choice, and cultural, historical and gender issues) emerged from the data corpus. Although the extent and consistency of these understandings call for further investigation, the results may inform the development of a trauma‐informed clinical supervision model for nurses and midwives. Further research may help clarify trauma‐informed supervision and inform pedagogical strategies.

## 1 Introduction

Nurses and midwives are exposed to a range of occupational stressors—including workplace aggression, patient death, bullying, moral injury, insufficient education, inadequate staffing, suboptimal remuneration, gender‐based exclusion and racism—that may precipitate trauma responses. This review examines the critical role of trauma‐informed clinical supervision for nurses and midwives, with a focus on workforce retention, well‐being and organisational culture.

Extensive research highlights persistent shortages in nursing and midwifery (Boniol et al. 2022; Pressley et al. 2022; Tamata and Mohammadnezhad 2022). These workforce deficits contribute to increased intentions to leave the profession, with global demand for nurses projected to reach between 4.1 (World Health Organization 2025) and 4.5 million by 2030 (Boniol et al. 2022). Staffing gaps are associated with psychological distress, including burnout and compassion fatigue (Isobel and Thomas 2022; Maben et al. 2026; Usher and Jackson 2023). Exposure to trauma elevates the risk of vicarious traumatisation and attrition, and inadequate responses to these problems may undermine organisational safety and trust (Berring et al. 2024).

Trauma‐Informed Care (TIC) addresses such challenges by building trauma awareness into healthcare organisations (Substance Abuse and Mental Health Services Administration [SAMHSA] 2014). TIC encourages compassionate care and aims to prevent re‐traumatisation, supporting patient autonomy. Its strengths‐based framework recognises the effects of trauma on individuals, professionals and communities. TIC also focuses on organisational culture, not just symptom management and treatment.

The TIC framework is based on six core principles: Safety, Trustworthiness and Transparency, Peer Support, Collaboration and Mutuality, Empowerment, Voice and Choice, and Cultural, Historical and Gender Issues (Substance Abuse and Mental Health Services Administration 2014). These principles provide support for healthcare professionals affected by trauma and address trauma's impact across multiple levels (Berring et al. 2024). Comprehensive implementation of all six principles facilitates broad support, maintains professional well‐being, enhances cultural sensitivity, prevents re‐traumatisation and promotes organisational change. Berring et al. (2024) emphasise that only the full integration of all of these principles enables TIC to achieve its intended outcomes.

Expanding upon trauma‐informed strategies, Butterworth (2022) identifies clinical supervision as a structured method to support practitioner development and maintain professional quality of life in the context of workplace trauma. Clinical supervision has also been demonstrated to enhance collegiality among mental health nurses (Hamilton et al. 2024). However, scepticism, negative perceptions and inadequate organisational support may impede its effective implementation (Masamha et al. 2022; Saab et al. 2021; Tulleners et al. 2024).

Transparency in the selection and implementation of clinical supervision has been shown to improve outcomes (Atashi et al. 2024; Rothwell et al. 2021). Although many nurses are assigned supervisors and supervision modalities without input, those who can select their own supervisors report greater professional empowerment (Cook et al. 2020; O'Neill et al. 2023).

Cultural humility represents a critical component of clinical supervision, yet it is often insufficient, particularly for nurses in remote regions of Australia (Hildebrand et al. 2023). In colonised countries, recommendations emphasise integrating First Nations knowledge and prioritising cultural safety in clinical supervision (Sorby et al. 2025). Enhancing supervisors' multicultural competency may address microaggressions in cross‐racial supervision and support the retention of international nurses (Pressley et al. 2022; Sukumaran et al. 2025).

Recent evidence indicates that nurses and midwives frequently have histories of adverse childhood experiences, trauma and stress (Wolotira 2023). Individuals with these backgrounds, as well as those employed in high‐acuity roles, are at increased risk of workplace stress and associated mental health conditions, including anxiety, depression, post‐traumatic stress disorder (PTSD), compassion fatigue and burnout (Lown et al. 2023).

Clinical supervision in nursing and midwifery has undergone significant evolution. In response to the COVID‐19 pandemic, an increasing number of organisations adopted restorative supervision models (Baldwin et al. 2022; Carter 2022; Francis 2024; Klages et al. 2024; Wade 2023). These models, originally developed to support midwives following bereavement (Wallbank 2016), employ Professional Quality of Life (ProQOL) strategies to enhance compassion satisfaction and reduce burnout (Baldwin et al. 2022; Stamm 2009).

Trauma‐informed clinical supervision is well established in allied health as a means to reduce burnout among professionals working with trauma‐affected clients (Bledsoe et al. 2022; Borders et al. 2023; Radis 2019; Varghese et al. 2018). However, this approach remains underexplored within the nursing profession (McGarva et al. 2024). The present review addresses this gap by synthesising existing research and providing evidence‐based insights to inform the development of trauma‐informed clinical supervision for nurses and midwives, with particular attention to workforce retention. This study aimed to consolidate evidence from the scholarly literature on trauma‐informed clinical supervision in nursing and midwifery. To guide this focus, it addresses the question: ‘Can the six principles of trauma‐informed care be integrated into clinical supervision practices for nurses and midwives?’

## 2 Method

This integrative literature review adheres to the methodological guidance established by Whittemore and Knafl (2005), a framework widely recognised within nursing for its contribution to the advancement of nursing and midwifery scholarship. The integrative review methodology is particularly valued for its utility in informing future research, clinical practice and policy development (Whittemore and Knafl 2005). By incorporating both empirical and theoretical sources, this approach facilitates a nuanced and comprehensive examination of topics pertinent to the research question (Whittemore and Knafl 2005). To ensure our findings remain salient to the context of clinical supervision for nurses and midwives, we adopted directed qualitative content analysis to provide descriptive knowledge and understandings of the phenomenon under study. This analytic approach provides the flexibility necessary to critically interrogate and reflect upon our own personal and theoretical assumptions, as well as our positionality as trauma‐informed health‐care professionals.

### 2.1 Search Strategy

The review is reported using the Preferred Reporting Items for Systematic Reviews and Meta‐Analysis PRISMA 2020 statement (Page et al. 2021). Literature was first identified by title. Next, abstracts were screened. Articles that met the inclusion criteria were sent for full‐text review. Eligible articles were then included in this review. Search terms were developed in collaboration with a University Librarian. Databases searched between November and December 2025 included PubMed, CINAHL, EMBASE, PsycINFO and Google Scholar. To improve comprehensiveness and reduce publication bias, grey literature was searched using ProQuest and Google Scholar. The first 50 sources identified were screened. Grey literature, such as theses, discursive articles and editorials, was included if it addressed trauma‐informed principles or clinical supervision for nurses or midwives. The search strategy also incorporated the terms ‘nurs*’ OR ‘midwi*’ OR ‘psychological trauma’ OR ‘trauma‐informed’ AND ‘clinical supervision’. Keywords and Medical Subject Headings (MeSH) were adapted for each database. Truncation was applied as appropriate, according to each database's syntax. Table 1 presents initial search results.

**TABLE 1:** Overview of the initial search results.

Source	Search area	Coverage	Keywords	Records
CINAHL	Key word	2021–2025	nurs* AND OR midwi* AND supervis* AND trauma‐informed	124
MEDLINE	Key word	2021–2025	nurs* AND OR midwi* AND supervis* AND trauma‐informed	118
ProQuest	Key word	2021–2025	nurs* AND OR midwi* AND supervis* AND trauma‐informed	50
Embase	Key word	2021–2025	nurs* AND OR midwi* AND supervis* AND trauma‐informed	38
PubMed	Key word	2021–2025	nurs* AND OR midwi* AND supervis* AND trauma‐informed	10
Google Scholar	Title	2021–2025	nurse OR midwife AND supervision AND trauma‐informed	25
Hand search	Title/abstract	2021–2025	nurse OR midwife AND supervision AND trauma‐informed	29
Total	394			

### 2.2 Inclusion and Exclusion Criteria

The inclusion criteria for this integrative literature review were developed to address the central research question: whether the six principles of trauma‐informed care can be integrated into clinical supervision practices for nurses and midwives. To ensure methodological rigour and transparency, studies were considered eligible if they were published in English between January 1, 2021 and December 30, 2025, and utilised qualitative, quantitative or mixed‐methods research designs. Eligible studies were required to engage with trauma‐informed care principles as outlined by the Substance Abuse and Mental Health Services Administration (2014), including safety, trustworthiness and transparency, peer support, collaboration and mutuality, empowerment, voice and choice, and cultural, historical and gender considerations. Studies also needed to include nurses or midwives within their participant populations, clinical contexts or data sets, either as the primary focus or as part of a broader health or social care workforce relevant to nursing or midwifery practice. Relevance to clinical supervision was a required criterion, and for studies involving mixed professional groups, inclusion was determined by the applicability of the context, participant group or findings to nursing or midwifery. Additionally, grey literature, such as theses, discursive articles and editorials, was included if it addressed trauma‐informed care principles or clinical supervision relevant to nursing or midwifery. This approach was intended to support a focused synthesis of contemporary evidence in the field.

### 2.3 Screening

One author conducted each screening stage. Co‐authors reviewed selections to improve rigour. During the initial screening phase, 182 records were excluded due to ineligibility or duplication. Of the 249 records initially screened by title, 114 were excluded for failing to meet the established eligibility criteria. Subsequent screening of titles and abstracts for 105 records resulted in the removal of an additional 72 records deemed irrelevant. Full‐text assessment of the remaining 33 records led to a further 26 exclusions: five did not address clinical supervision for nurses or midwives, and 11 did not reference the six trauma‐informed care principles. Figure 1 provides an overview of the review process.

**FIGURE 1:**
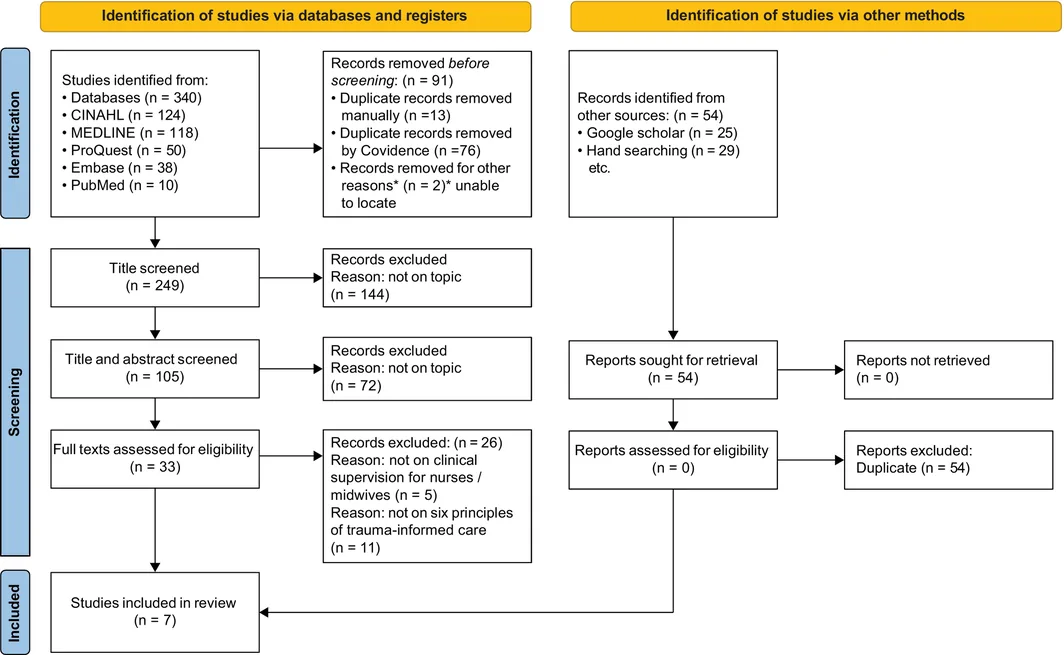
PRISMA flow chart of systematic literature search (Page et al. 2021).

### 2.4 Analysis

A directed qualitative content analysis was conducted to examine the dataset, using the principles of trauma‐informed care outlined by the Substance Abuse and Mental Health Services Administration (SAMHSA 2014) as the theoretical framework. Following established methodological guidance (Hsieh and Shannon 2005; Humble and Mozelius 2022), six a priori codes were used: (1) safety; (2) peer support; (3) trustworthiness and transparency; (4) collaboration and mutuality; (5) empowerment, voice and choice; and (6) cultural, historical and gender considerations. A structured codebook was created to ensure consistent application of these codes to textual data from the seven included studies. The analysis included quantification through frequency counts of coded segments and categorisation of the occurrence and distribution of each principle. This allowed an objective assessment of how these principles were described and operationalised within clinical supervision. Coding categories were refined as needed while staying anchored in the original trauma‐informed care framework. The research team collaboratively developed and modified the codebook to maintain methodological rigour. This analytical approach provides insights to inform the development of a trauma‐informed care framework for clinical supervision in nursing and midwifery.

### 2.5 Assessment of Methodological Quality

Whittemore and Knafl (2005) underscore the inherent challenges associated with quality appraisal in heterogeneous bodies of literature. Due to the high degree of heterogeneity in the literature identified and as all literature that satisfied the inclusion criteria, including grey literature, was retained, formal quality appraisal was not undertaken. The final review is therefore narrative in nature.

## 3 Results

See Table 2 for an overview of the studies identified in the final review. This review included seven scholarly articles: four from the United States, one from New Zealand, one from the United Kingdom and one from Australia. Three were academic theses: a doctoral dissertation from the United Kingdom exploring therapists' experiences in managing secondary traumatic stress (Copestake 2021), a doctoral dissertation from the United States examining nurses' perceptions and experiences related to trauma‐informed care (Hayter et al. 2025), and a master's dissertation from Australia examining trauma‐informed care education for midwives (Long 2024). Another article outlined the development of the Trauma‐Informed Supervision Framework (TISF) within addiction treatment settings in the United States (Koury et al. 2025). The New Zealand article provided an integrative review and thematic analysis of trauma‐informed supervision for nurses (McGarva et al. 2024). One article considered the delivery of gender‐sensitive and responsive trauma‐informed psychiatric care by nurses to cisgender female patients in mental health services in the United States (Hauenstein and Schimmels 2024). The final paper analysed organisational strategies in the United States for implementing trauma‐informed approaches to address nursing burnout (Dawson‐Rose et al. 2023).

**TABLE 2:** Summary of included literature.

Article title, author, year	Design	Country	Population	Study aims	Cites six principles of trauma‐informed care
You're going to connect with the stories‐A qualitative analysis of secondary traumatic stress and the role of clinical supervision (Copestake 2021)	Thesis	UK	Therapists (including nurses)	To explore the experiences of therapists in managing secondary traumatic stress and vicarious trauma in clinical practice	Yes
Using a trauma‐informed approach to address burnout in nursing. What an organisation can accomplish (Dawson‐Rose et al. 2023)	Discussion	USA	Nurses	To explore how a trauma‐informed approach can be implemented in an organisation to address burnout	Yes
Providing gender sensitive and responsive trauma‐informed nursing care (Hauenstein and Schimmels 2024)	Literature review	USA	Nurses	To examine how gender identity, subordination and trauma history affect psychiatric nurses' ability to provide effective care	Yes
Factors associated with nurses' perceptions and practices of trauma‐informed care (Hayter et al. 2025)	Thesis	USA	Nurses	To explore individual and organisational factors associated with nurses' perceptions and practices of Trauma‐Informed Care (TIC)	Yes
Trauma‐informed supervision: A practical framework for addiction setting (Koury et al. 2025)	Discussion	USA	Addictions professionals (including nurses)	To discuss the Trauma‐Informed Supervision Framework (TISF) as a practical framework for supervisors in addiction settings	Yes
Strengthening connections: Trauma informed care education for midwives (Long 2024)	Thesis	AUS	Midwives	To evaluate the impact of trauma‐informed care (TIC) education on midwives' attitudes towards providing trauma‐informed care	Yes
Insights towards trauma‐informed nursing supervision: An integrative literature review and thematic analysis (McGarva et al. 2024)	Literature review	NZ	Nurses	To explore the literature related to trauma‐informed supervision in nursing	Yes

All seven studies reviewed referenced the six principles of trauma‐informed care and clinical supervision, though not all detailed their implementation within specific supervisory modalities. Among the seven studies examined, three addressed safety, trustworthiness and transparency; two discussed peer support; three considered collaboration and mutuality; two examined empowerment, voice and choice; and two discussed cultural, historical and gender‐related considerations. Table 3 presents the five studies that referenced at least one of the six principles.

**TABLE 3:** Inclusion of six principles of trauma‐informed care.

Article title, author, year	Safety in clinical supervision	Trustworthiness and transparency in clinical supervision	Peer support in clinical supervision	Collaboration and mutuality in clinical supervision	Empowerment, voice and choice in clinical supervision	Cultural, historical and gender issues in clinical supervision
You're going to connect with the stories‐A qualitative analysis of secondary traumatic stress and the role of clinical supervision (Copestake 2021)	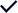	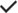	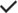			
Using a trauma‐informed approach to address burnout in nursing. What an organisation can accomplish (Dawson‐Rose et al. 2023)			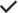	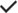		
Providing gender sensitive and responsive trauma‐informed nursing care (Hauenstein and Schimmels 2024)						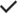
Trauma‐informed supervision: A practical framework for addiction setting (Koury et al. 2025)	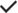	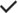		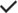	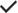	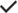
Insights towards trauma‐informed nursing supervision: An integrative literature review and thematic analysis (McGarva et al. 2024)	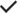	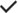		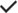	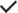	

This finding aligns the observations of Berring et al. (2024), who reported that although many studies reference SAMHSA's six core principles, their practical application is often inconsistent, with some studies omitting them entirely or addressing them only partially. Berring et al. (2024) further emphasise the importance of a clearly defined concept of trauma‐informed care and highlight the need for broader international consensus on the operationalisation of the six principles of trauma‐informed care.

During data extraction and mapping, two studies failed to operationalise any of the six principles within their supervision activities, despite referencing the six SAMHSA principles within their papers. One study focused on factors influencing nurses' perceptions and practices regarding trauma‐informed care, whereas the other emphasised enhancing professional connections through trauma‐informed education for midwives (Hayter et al. 2025; Long 2024).

Hayter et al. (2025) investigated the application of the six principles of trauma‐informed care (TIC) within healthcare organisational structures and leadership but did not address their integration within clinical supervision. The study recommended systemic changes to support nursing professionals and promote a trauma‐informed healthcare environment, highlighting the need for targeted training programmes and interventions at both individual and organisational levels. The authors identified a lack of clear implementation standards, which contribute to ambiguity in the operationalisation of ‘trauma‐informed’ practices. They also noted that most TIC frameworks include screening, collaborative practices, workforce development (including training, governance and policy formation), prevention of re‐traumatisation and secondary traumatic stress, cultural sensitivity and programme monitoring and evaluation.

The subsequent study examined the integration of the six principles of trauma‐informed care into midwifery education (Long 2024). Midwives who participated in a 2‐day trauma‐informed care programme showed statistically significant improvements in attitudes towards trauma‐informed care compared to non‐participants. The intervention was associated with positive outcomes across the domains of the Attitudes Related to Trauma‐Informed Care (ARTIC‐35) scale, including understanding of problem behaviours, responses to such behaviours, on‐the‐job behaviour, self‐efficacy, and responses to work‐related trauma. Although participants found trauma‐informed care education beneficial, they reported receiving more collegial than administrative support and continued to encounter organisational barriers. The authors noted an ongoing need for support mechanisms to address secondary trauma.

Although Hayter et al. (2025) and Long (2024) did not operationalise the six principles of trauma‐informed care within clinical supervision, leaders in clinical supervision may draw upon their existing frameworks and educational interventions to establish standards for trauma‐informed care in clinical supervision for nurses and midwives. The development of such standards could support more consistent implementation of the six principles of trauma‐informed care within clinical supervision.

The next section details how each principle was incorporated into clinical supervision, with definitions adapted from Sandra Bloom, founder of the Sanctuary Model, whose work informs interventions for both care recipients and healthcare professionals affected by trauma (Bloom et al. 2013).

### 3.1 Safety

Bloom et al. (2013) maintain that establishing a culture of safety is a meaningful objective in clinical supervision, where a supportive, non‐judgmental environment underpins supervisees' psychological well‐being. Safety is emphasised in the literature, with several studies identifying it as a recurring theme (Copestake 2021; Koury et al. 2025; McGarva et al. 2024). The evidence indicates that emotionally safe environments can promote self‐reflection, emotional validation, and the normalisation of trauma responses, which may contribute to improved professional quality of life and resilience (Copestake 2021; Koury et al. 2025; McGarva et al. 2024). Moreover, the supervisor's attentiveness to their supervisees' psychological health is associated with the development of psychological safety (Koury et al. 2025; McGarva et al. 2024). The literature identifies supervisory practices such as facilitating reflective processes, validating the supervisees' emotional experiences, normalising trauma responses, engaging empathetically, safeguarding confidentiality and refraining from providing immediate solutions. These practices may create a culture of safety in clinical supervision for nurses and midwives (Copestake 2021; Koury et al. 2025; McGarva et al. 2024). In addition, the therapeutic use of self by supervisors, including sensitivity to communication preferences, observation of the supervisee's responses, maintenance of professional composure, and the use of inclusive language, has been identified as relevant to psychological and social safety within supervisory relationships (Koury et al. 2025).

### 3.2 Transparency and Trustworthiness

The development of a culture of trustworthiness and transparency within organisations is aided by an ongoing commitment to open, honest and compassionate communication (Bloom et al. 2013). The literature, including works by Copestake (2021), Koury et al. (2025) and McGarva et al. (2024), indicates that transparency and trustworthiness are important elements of clinical supervision for nurses and midwives. These sources further suggest that supervisory relationships, including regular meetings, can support trust and alleviate supervisee anxiety. The literature also notes that encouraging vulnerability and sharing relevant experiences, particularly those related to trauma‐informed practice, can contribute to transparency, inclusion and mutual respect.

### 3.3 Peer Support

Peer support within clinical supervision is characterised by collaborative and reciprocal engagement, fostering mutual accountability and shared responsibility (Bloom et al. 2013). The literature demonstrates that peer support in clinical supervision strengthens professional communities by promoting collaboration and mutual aid (Copestake 2021; Dawson‐Rose et al. 2023). Group supervision is reported to facilitate collective learning, the exchange of professional experiences and the development of resilience. It may also reduce professional isolation and enhance professional quality of life (Dawson‐Rose et al. 2023). Additionally, informal peer support and debriefing offer opportunities to share coping strategies, provide mutual support, and promote emotional regulation, thereby further reducing professional isolation (Copestake 2021).

### 3.4 Collaboration and Mutuality

Bloom et al. (2013) identify collaboration and mutuality as key components of relationships embodied by shared power, partnership and equality. This perspective is endorsed by the literature (Dawson‐Rose et al. 2023; Koury et al. 2025; McGarva et al. 2024). Within clinical supervision, Dawson‐Rose et al. (2023) and Koury et al. (2025) suggest that collaboration and mutuality lead to active participation in organisational decision‐making and the establishment of shared goals and plans, both of which can influence professional quality of life and well‐being. Koury et al. (2025) also note the salience of addressing power imbalances, culture, privilege, trauma, inclusivity and mutual respect within supervisory relationships. The findings of Koury et al. (2025) and McGarva et al. (2024) highlight the value of cultivating mutual respect, recognising the diversity of expertise and experiences, and promoting candid communication in supervision. McGarva et al. (2024) further discuss strategies such as co‐constructing knowledge, developing trauma‐informed care skills, and taking part in collaborative reflection. Both Koury et al. (2025) and McGarva et al. (2024) recommend modelling collaborative practices, using non‐discriminatory language, and addressing cultural and power dynamics. Together, these authors offer perspectives on fostering mutual understanding in clinical supervision for nurses and midwives.

### 3.5 Empowerment, Voice and Choice

Bloom et al. (2013) describe empowerment, voice and choice in supervision as crucial for establishing a trauma‐informed, collaborative environment that supports growth and recovery. The reviewed literature recognises empowerment, voice and choice as central to effective trauma‐informed clinical supervision. Empowerment fosters autonomy and self‐determination, encouraging nurses and midwives to guide their own professional development and decision‐making (Koury et al. 2025). Voice ensures supervisees are heard, their perspectives are respected, and their feedback is integrated into supervisory practices (McGarva et al. 2024). Choice is reflected in joint decision‐making and flexible supervision agreements that consider individual preferences and needs (Koury et al. 2025). Together, these elements suggest that supervision grounded in empowerment, voice and choice enhances skill development and confidence while creating a supportive, collaborative environment.

### 3.6 Cultural, Historical and Gender Issues

An understanding of cultural, historical and gender‐related factors enables health professionals to interpret how these factors shape people's and communities' experiences (Bloom et al. 2013). The reviewed studies indicate that clinical supervision has an important role in cultivating this understanding, particularly when trauma‐informed care (TIC) principles are integrated into practice (Hauenstein and Schimmels 2024; Koury et al. 2025). In this context, transparency involves the explicit engagement of supervisors and supervisees in discussions and critical reflection on these factors, rather than leaving them implicit and unsaid. Supervisors augment professional development by intentionally addressing intersecting identities—such as race, gender, age and ability—and by inviting critical dialogue about how these identities shape trauma experiences and interpersonal relations (Koury et al. 2025). The authors suggest that gender self‐awareness can be promoted using reflection and mindfulness‐based strategies, including the RAIN technique (Recognise, Allow, Investigate, Nurture) (Hauenstein and Schimmels 2024). Furthermore, committing to diversity, equity and inclusion (DEI) and sustaining open conversations regarding race, culture, privilege, pronouns and relevant sociocultural events may support a more inclusive supervisory environment (Koury et al. 2025).

## 4 Discussion

This review examined the systematic integration of the six principles of trauma‐informed care into clinical supervision for nurses and midwives. Despite widespread recognition in healthcare, conceptualisation and application of these principles into practice remain inconsistent (Berring et al. 2024). Existing studies emphasise different aspects—such as safety, trust, peer support or culture—resulting in heterogeneous practices and an absence of a unified framework. Such variability likely reflects methodological and contextual differences across settings, perpetuating debate between standardised and adaptive models. The lack of explicit supervisory frameworks and selective application of trauma‐informed principles underscores the need for further research. Systematic approaches may better bridge theory and practice, supporting comprehensive integration.

Findings indicate challenges in integrating the six principles of trauma‐informed care into clinical supervision. Each principle presents distinct obstacles. For instance, implementation of safety is often inconsistent, reflecting variable supervisory competence and institutional support, and contributing to a gap between policy and practice (Copestake 2021; Koury et al. 2025; McGarva et al. 2024). Fostering trustworthiness and transparency may be achieved through structured meetings, yet sustaining open communication remains difficult in diverse clinical environments. Peer support, while potentially beneficial, is sometimes insufficiently developed and may inadvertently reinforce hierarchy (Copestake 2021; Dawson‐Rose et al. 2023). Efforts to enhance collaboration and mutuality are linked to well‐being, but entrenched power dynamics may limit genuine partnership, highlighting the need for intentional inclusivity (Dawson‐Rose et al. 2023; Koury et al. 2025; McGarva et al. 2024). Although empowerment, voice and choice are supported in principle, organisational cultures favouring acquiescence over engagement may impede their realisation (Koury et al. 2025; McGarva et al. 2024). Achieving meaningful empowerment may require robust feedback mechanisms and supervisory flexibility (Koury et al. 2025). Finally, although cultural, historical and gender‐related factors are acknowledged, institutional responses are often limited, indicating the need for more sustained, reflective and embedded equity strategies in trauma‐informed supervision (Hauenstein and Schimmels 2024; Koury et al. 2025).

In summary, this review identifies key barriers and highlights the potential of systematic, context‐sensitive approaches for integrating trauma‐informed principles into clinical supervision for nurses and midwives. Although trauma‐informed clinical supervision is important, ongoing workforce shortages are likely to continue to challenge workplace well‐being and the quality of care.

## 5 Relevance for Clinical Practice

This integrative literature review suggests that trauma‐informed clinical supervision for nurses and midwives, informed by the six principles of trauma‐informed care, offers a framework for future application, development and empirical evaluation within nursing and midwifery contexts. By supporting practitioners in recognising and addressing the varied effects of trauma, including professional and vicarious experiences, trauma‐informed clinical supervision may improve reflective practice, critical thinking and ethical leadership, which are valued aspects of nursing and midwifery scholarship. Engagement in this model of clinical supervision may also assist practitioners in examining organisational power dynamics and workplace challenges, such as aggression, patient mortality, bullying, moral injury, resource limitations, understaffing, inequitable remuneration and gender inequity. Integrating these considerations into academic discourse could contribute to the development of research‐based strategies for workforce sustainability and retention, supporting the profession's ongoing commitment to advocacy and improvement.

## 6 Conclusion

These preliminary findings suggest the need for further empirical inquiry. Emerging evidence indicates that trauma‐informed clinical supervision could be developed for nurses and midwives, particularly when coupled with educational programmes to operationalise the six principles of trauma‐informed care. The integration of vicarious trauma and self‐care modules into nursing and midwifery curricula may also be beneficial. Continued research could inform interventions to improve workplace wellbeing, retention and patient outcomes.

## Funding

The authors have nothing to report.

## Ethics Statement

The authors have nothing to report.

## Consent

The authors have nothing to report.

## Conflicts of Interest

The first author provides and teaches clinical supervision. The authors declare no conflicts of interest.

## Data Availability

All data are available within the article.
